# Development of a novel immune-related lncRNA signature as a prognostic classifier for endometrial carcinoma

**DOI:** 10.7150/ijbs.51207

**Published:** 2021-01-01

**Authors:** Jinhui Liu, Jie Mei, Yichun Wang, Xucheng Chen, Jiadong Pan, Laigen Tong, Yan Zhang

**Affiliations:** 1 Department of Gynecology, The First Affiliated Hospital of Nanjing Medical University, Nanjing 210029, Jiangsu, China.; 2 Wuxi School of Clinical Medicine, Nanjing Medical University, Wuxi 214023, Jiangsu, China.; 3 Department of Urology, The First Affiliated Hospital of Nanjing Medical University, Nanjing 210029, Jiangsu, China.; 4 College of Pharmacy, Nanjing Medical University, Nanjing 211166, Jiangsu, China.; 5 Department of Hematology, Yixing People's Hospital, The Affiliated Hospital of Jiangsu University, Yixing 214200, Jiangsu, China.; 6 Department of Gynecology and Obstetrics, Wuxi Maternal and Child Health Hospital, the Affiliated Hospital of Nanjing Medical University, Wuxi 214000, Jiangsu, China.

**Keywords:** immune-related lncRNA, endometrial carcinoma, signature, bioinformatics

## Abstract

Endometrial carcinoma (EnCa) is one of the deadliest gynecological malignancies. The purpose of the current study was to develop an immune-related lncRNA prognostic signature for EnCa. In the current research, a series of systematic bioinformatics analyses were conducted to develop a novel immune-related lncRNA prognostic signature to predict disease-free survival (DFS) and response to immunotherapy and chemotherapy in EnCa. Based on the newly developed signature, immune status and mutational loading between high‑ and low‑risk groups were also compared. A novel 13-lncRNA signature associated with DFS of EnCa patients was ultimately developed using systematic bioinformatics analyses. The prognostic signature allowed us to distinguish samples with different risks with relatively high accuracy. In addition, univariate and multivariate Cox regression analyses confirmed that the signature was an independent factor for predicting DFS in EnCa. Moreover, a predictive nomogram combined with the risk signature and clinical stage was constructed to accurately predict 1-, 2-, 3-, and 5-year DFS of EnCa patients. Additionally, EnCa patients with different levels of risk had markedly different immune statuses and mutational loadings. Our findings indicate that the immune-related 13-lncRNA signature is a promising classifier for prognosis and response to immunotherapy and chemotherapy for EnCa.

## Introduction

Endometrial carcinoma (EnCa), which originates from glandular epithelial cells of the endometrium, is the third most common type of malignant tumor in the female reproductive system worldwide 1. According to the statistical data published by the International Agency for Research on Cancer, EnCa comprises 4.8% of cancers and has a tumor-related mortality rate of 2.1% among females worldwide 2. According to the International Federation of Gynecology and Obstetrics (FIGO) criteria, EnCa is divided into two subtypes based on endocrine and clinical features: type I carcinomas, which are related to endometrial hyperplasia and are estrogen dependent, and type II carcinomas, which are related to endometrial atrophy and are estrogen independent 3. Although continual efforts have been made to overcome one difficulty after another in the long-term quest to treat EnCa, the prognosis of patients with advanced-stage disease is unsatisfactory. Therefore, it is of great significance to identify novel strategies to accurately predict the prognosis of EnCa.

Recently, it was proven that immunotherapies can be novel therapeutic strategies for EnCa and that the local immune status affects tumor progression and determines the response to immunotherapies 4, 5. Long noncoding RNAs (lncRNAs) have a wide range of functional activities 6, 7, and dysregulation of critical lncRNAs may contribute to the carcinogenesis and progression of EnCa 8, 9. Moreover, lncRNAs have been identified as crucial nodes in regulating tumor immunity in multiple cancers 10, 11. For example, lncMX1-215 markedly downregulates IFNα-induced programmed cell death 1 ligand 1 (PD-L1) and galectin-9 expression by interrupting GCN5/H3K27ac binding in head and neck squamous cell carcinoma 12. Therefore, dysregulation of immune-related lncRNAs may be identified as potential therapeutic targets and have prognostic value for EnCa patients.

In the current research, we developed a novel immune-related 13-lncRNA signature for EnCa patients that could accurately predict disease-free survival (DFS). In addition, based on the signature, the immune status and tumor mutation burden (TMB) in EnCa samples from different groups were distinctly varied. Notably, the findings obtained from this research may contribute to providing potential foundations for subsequent in-depth immune-related studies, to provide new insights into the individualized treatment of EnCa.

## Methods

### TCGA data acquisition

Level-3 mRNA expression profiles and clinical data were collected from 552 EnCa and 23 normal samples in the TCGA database (https://cancergenome.nih.gov). DFS was defined as the time from the starting point to disease recurrence or death due to disease progression, which was treated as the end point of observation in this research. After integration of clinical information, a total of 481 samples were defined as an entire set, which was randomly divided into a training set and a testing set. The testing set with 240 samples and the entire set were used to verify findings obtained from the training set with 241 samples. Both the entire mRNA profile data and the clinical information of the samples are publicly available.

### Definition of immune-related lncRNAs

First, immune-related genes were obtained from the Molecular Signatures Database v4.0 (Immune system process M13664, Immune response M19817, http://www.broadinstitute.org/gsea/msigdb/index.jsp) 13. In addition, the lncRNA profile was extracted from mRNA expression data on the basis of the GENCODE project (http://www.gencodegenes. org) 14. Then, a cohort of immune-related lncRNAs was identified according to Pearson correlation analysis, which was defined as lncRNA whose expression is correlated with any immune-related genes (at least one gene) (|R| > 0.5, *P* < 0.0001).

### Construction of immune-related the lncRNA signature for EnCa

These samples were randomly classified into the training cohort (n = 241) and the testing cohort (n = 240). The training cohort was used for prognostic signature construction, while the testing cohort and entire cohort were used for validation of the signature. Univariate Cox regression analysis was applied to screen prognosis-related genes from 45 immune-related lncRNAs with the criteria of *P* ≤ 0.05. LASSO Cox analysis was applied to identify lncRNAs most associated with DFS, and 10-round cross-validation was conducted to prevent overfitting. The filtered genes were entered into the multivariate Cox regression analysis, and a scoring model was built to predict DFS.

The risk score for each patient was then calculated using the following formula: risk score = -0.970896 * AL080317.2 + 0.173290 * ERICH6-AS1 - 1.091183 * AC016877.3 + 0.560300 * MCCC1-AS1 +0.153787 * AC120053.1 + 0.383809 * AC138932.5 - 0.690101 * ZNF433-AS1 - 0.123376 * SCARNA9 - 0.344099 * DBH-AS1 + 0.622167 * AL157932.1 + 0.236900 * AC073046.1 - 1.039016 * POC1B-AS1 + 0.428151 * AP003419.3. EnCa patients were divided into a high-risk group and a low-risk group at the cutoff of the median risk score. Univariate and multivariate Cox regression analysis was used to assess the prognostic values of risk score and other clinicopathological characteristics.

### Estimation of tumor-infiltrating immune cells

Transcriptome expression with standard annotation was uploaded to the CIBERSORT website (http://cibersort.stanford.edu/) 15, which was applied to assess the proportions of 22 types of tumor-infiltrating immune cells using the CIBERSORT algorithm following the standard procedure.

### Construction of mRNA-lncRNA coexpression networks

We analyzed the potential coexpression relationships of prognostic lncRNAs by Pearson's correlation analysis (|R| > 0.5, *P* < 0.0001). Then, the lncRNA-mRNA interaction network was visualized by Cytoscape software.

### Gene set enrichment analysis

In the entire TCGA cohort, samples of EnCa were divided into two groups according to the median risk score. In the assessment of potential biological functions related to risk score, gene set enrichment analysis (GSEA) was performed to detect associated signaling pathways between the two groups 16. KEGG terms enriched with FDR ≤ 0.05 were identified.

### Construction and validation of a predictive nomogram

Clinical stage and risk score, which had independent prognostic value, were incorporated to construct a nomogram to evaluate the probability of 1-, 2-, 3-, and 5-year DFS in EnCa. Validation of the nomogram was evaluated by calibration plot. The calibration curve of the nomogram was plotted to assess the nomogram-predicted probabilities against the actual rates.

### Mutation analysis

To compare the mutational loading between the two groups, the mutation annotation format (MAF) based on the TCGA cohort was functioned by the “maftools” package 17. In addition, TMB between the high- and low-risk groups was compared, and the correlation between TMB and risk score was also assessed.

### The immunophenoscore analysis

The immunophenoscore (IPS) of the tumor samples was calculated by analyzing the expression of genes in the following four major categories that determine immunogenicity: effector cells, immunosuppressive cells, MHC molecules, and immunomodulators 18. The IPS was calculated with a range of 0-10 based on the z-score for gene expression of representative cell types. The IPS for EnCa patients was downloaded from the Cancer Immunome Atlas (TCIA, https://tcia.at/home).

### Chemotherapeutic response prediction

In this study, the chemo-response to several drugs was predicted based on the online pharmaceutic database (Genomics of Drug Sensitibity in Cancer, GDSC), this database performed a large-scale drug screen incorporating detailed genomic analyses to systematically identify drug response biomarkers. This database screened >1000 genetically characterised human cancer cell lines with a wide range of anti-cancer therapeutics. The sensitivity patterns of the cell lines are correlated with extensive genomic data to identify genetic features that are predictive of sensitivity. This large collection of cell lines enables this database to capture much of the genomic heterogeneity that underlies human cancer, and which appears to play a critical role in determining the variable response of patients to treatment with specific agents. The half-maximal inhibitory concentration (IC50) of each drug was detected in plenty of cell lines. Besides, we also used the pRRophetic R package. This R package established statistical models from gene expression and drug sensitivity data in a very large panel of cancer cell lines, then applying these models to gene expression data from primary tumor tissues. With the help of the data from GDSC and pRRophetic R package, we predicted the IC50 in patients. For individual patients in the TCGA group, the exact treatment scheme is different, in this study; we predicted the chemo-response of several drugs based on the genomic gene expression pattern in high-risk and low-risk groups. All these predictions were based on the online pharmaceutic database rather than the actual treatment response. All parameters were set by the default values with removal of the batch effect of “combat” and tissue type of “allSoldTumors”, and duplicate gene expression was summarized as the mean value 19.

### Statistical analysis

All statistical analyses were applied by R version 3.6.3 (R package: pheatmap, ggplot2, rms, glmnet, forest plot, survminer, survival ROC, maftools, pRRophetic). For all analyses, two-tailed *P* ≤ 0.05 was regarded as statistically significant.

## Results

### Identification of immune-related lncRNAs

A total of 331 immune-related genes were collected from the Molecular Signatures Database. Then, 363 immune-related lncRNAs were identified according to Pearson correlation analysis (|R| > 0.5, *P* < 0.0001).

### Construction and validation of the immune-related 13-lncRNA signature

To construct an immune-related lncRNA prognostic signature, we used univariate Cox regression to screen the prognostic values of 363 lncRNAs. A total of 45 prognosis-associated lncRNAs were reserved with the criterion of *P* ≤ 0.05. Next, LASSO Cox analysis with ten-fold cross-validation was performed to further narrow the effective prognosis-associated lncRNAs (Fig. S1A, B). Subsequently, multivariate Cox proportional hazards regression analysis was used to build the prognostic signature. Finally, 13 immune-related lncRNAs were used to establish a prognostic signature (Fig. S1C). The risk score for each EnCa sample was calculated according to the expression levels of 13 immune-related lncRNAs and corresponding coefficients. Risk score = -0.970896 * AL080317.2 + 0.173290 * ERICH6-AS1 - 1.091183 * AC016877.3 + 0.560300 * MCCC1-AS1 + 0.153787 * AC120053.1 + 0.383809 * AC138932.5 - 0.690101 * ZNF433-AS1 - 0.123376 * SCARNA9 - 0.344099 * DBH-AS1 + 0.622167 * AL157932.1 + 0.236900 * AC073046.1 - 1.039016 * POC1B-AS1 + 0.428151 * AP003419.3.

**Figure 1:**
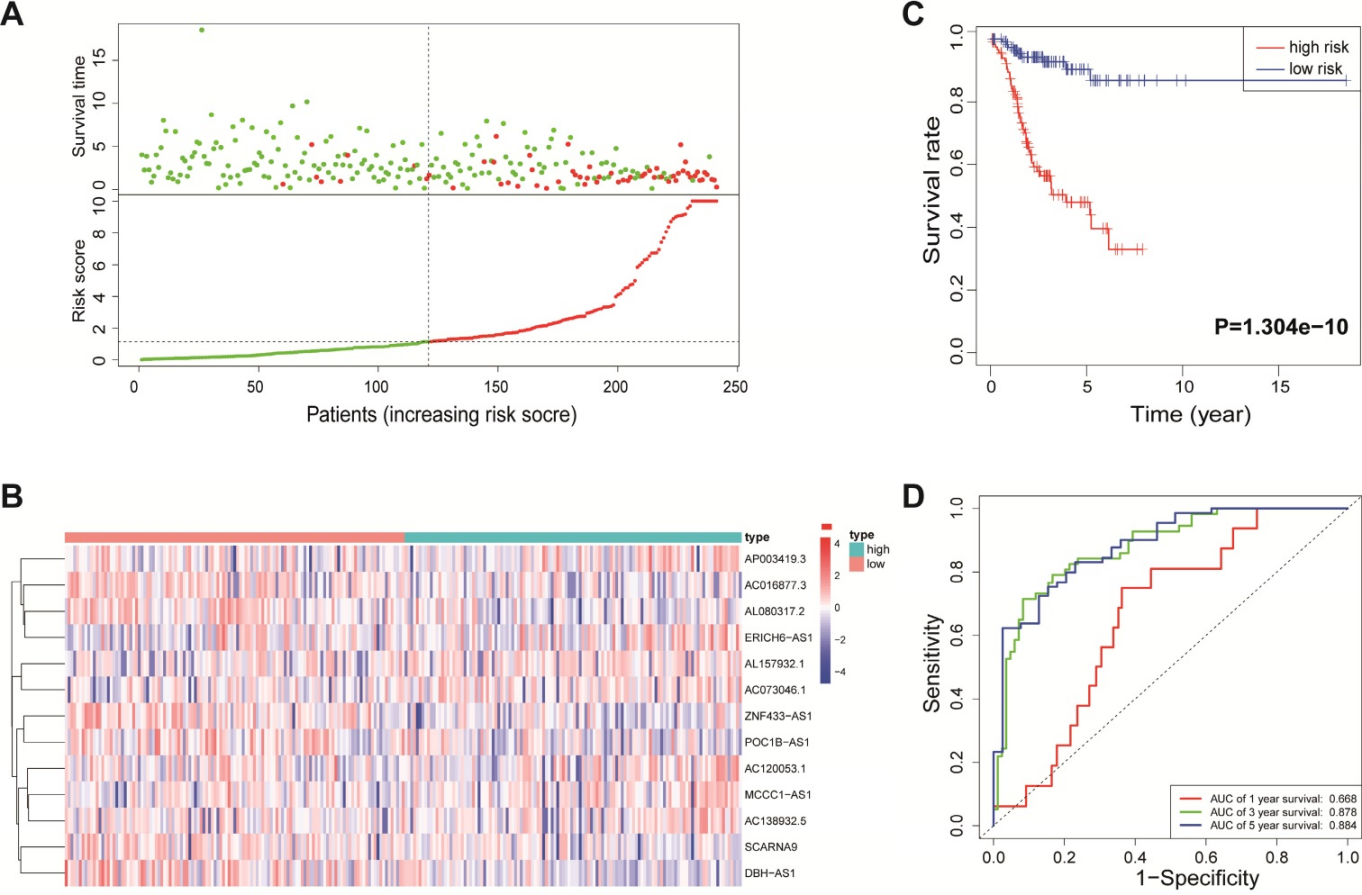
Identification of the immune-related lncRNA signature in the training cohort. (A) Survival status and risk score distribution in the high- and low-risk groups. Green dots: surviving patients; red dots: dead patients. (B) Expression patterns of 13 lncRNAs in high- and low-risk groups. (C) Kaplan-Meier curve analysis of DFS of EnCa patients in high- and low-risk groups. (D) Time-dependent ROC curve analysis.

**Figure 2:**
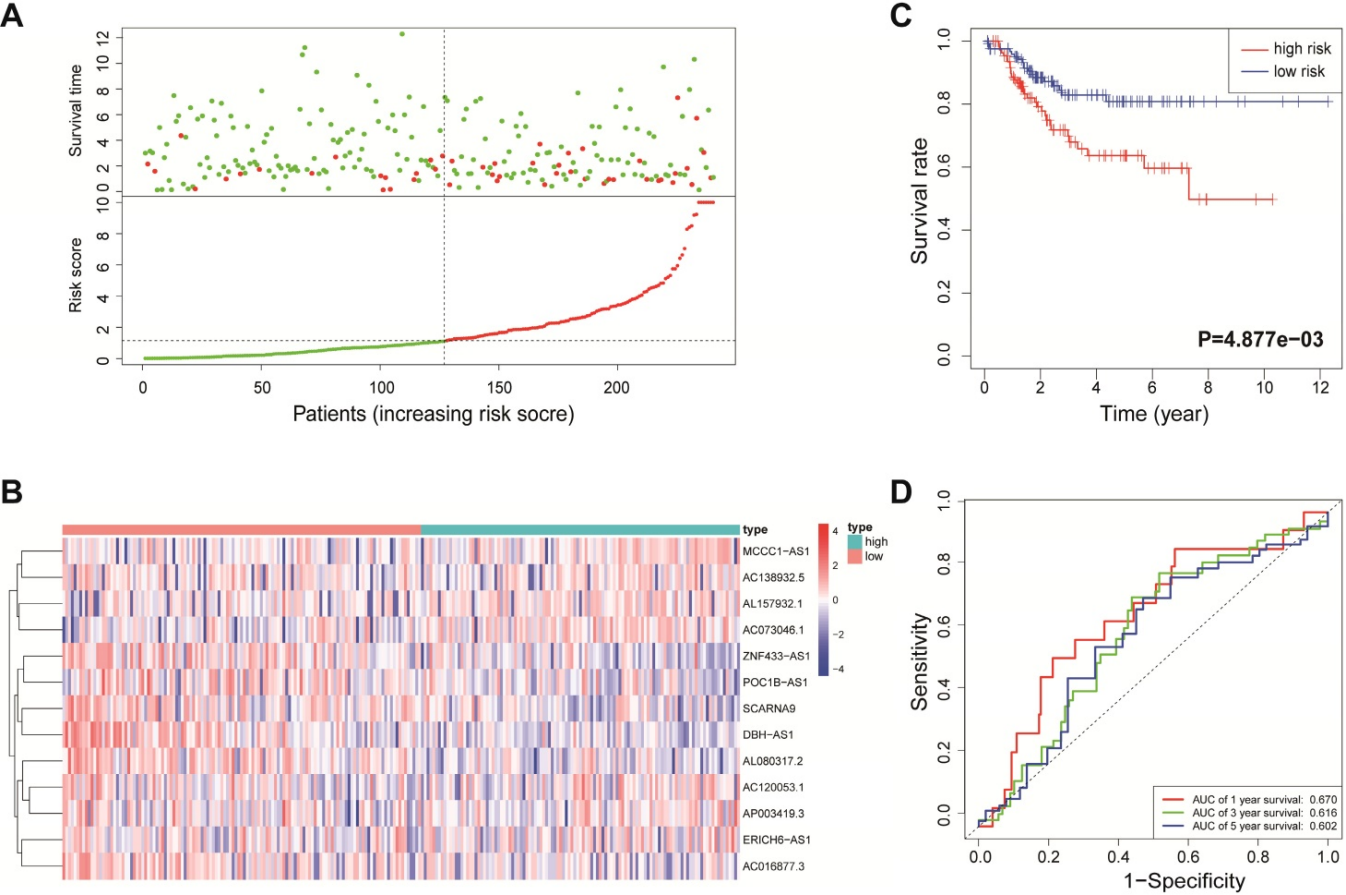
Validation of the immune-related lncRNA signature in the testing cohort. (A) Survival status and risk score distribution in the high- and low-risk groups. Green dots: surviving patients; red dots: dead patients. (B) Expression patterns of 13 lncRNAs in high- and low-risk groups. (C) Kaplan-Meier curve analysis of DFS of EnCa patients in high- and low-risk groups. (D) Time-dependent ROC curve analysis.

**Figure 3:**
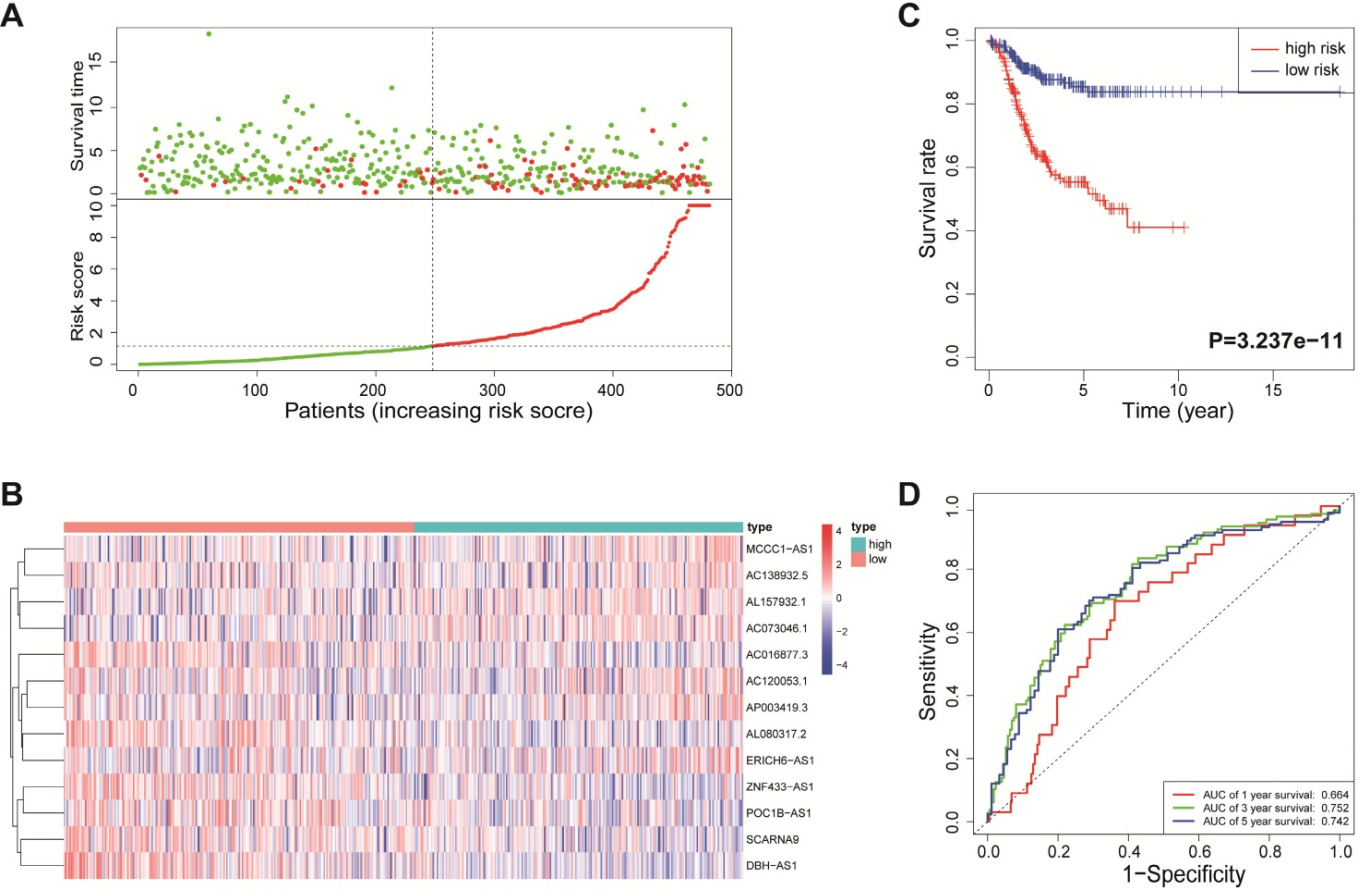
Validation of the immune-related lncRNA signature in the entire cohort. (A) Survival status and risk score distribution in the high- and low-risk groups. Green dots: surviving patients; red dots: dead patients. (B) Expression patterns of 13 lncRNAs in high- and low-risk groups. (C) Kaplan-Meier curve analysis of DFS of EnCa patients in high- and low-risk groups. (D) Time-dependent ROC curve analysis.

**Figure 4:**
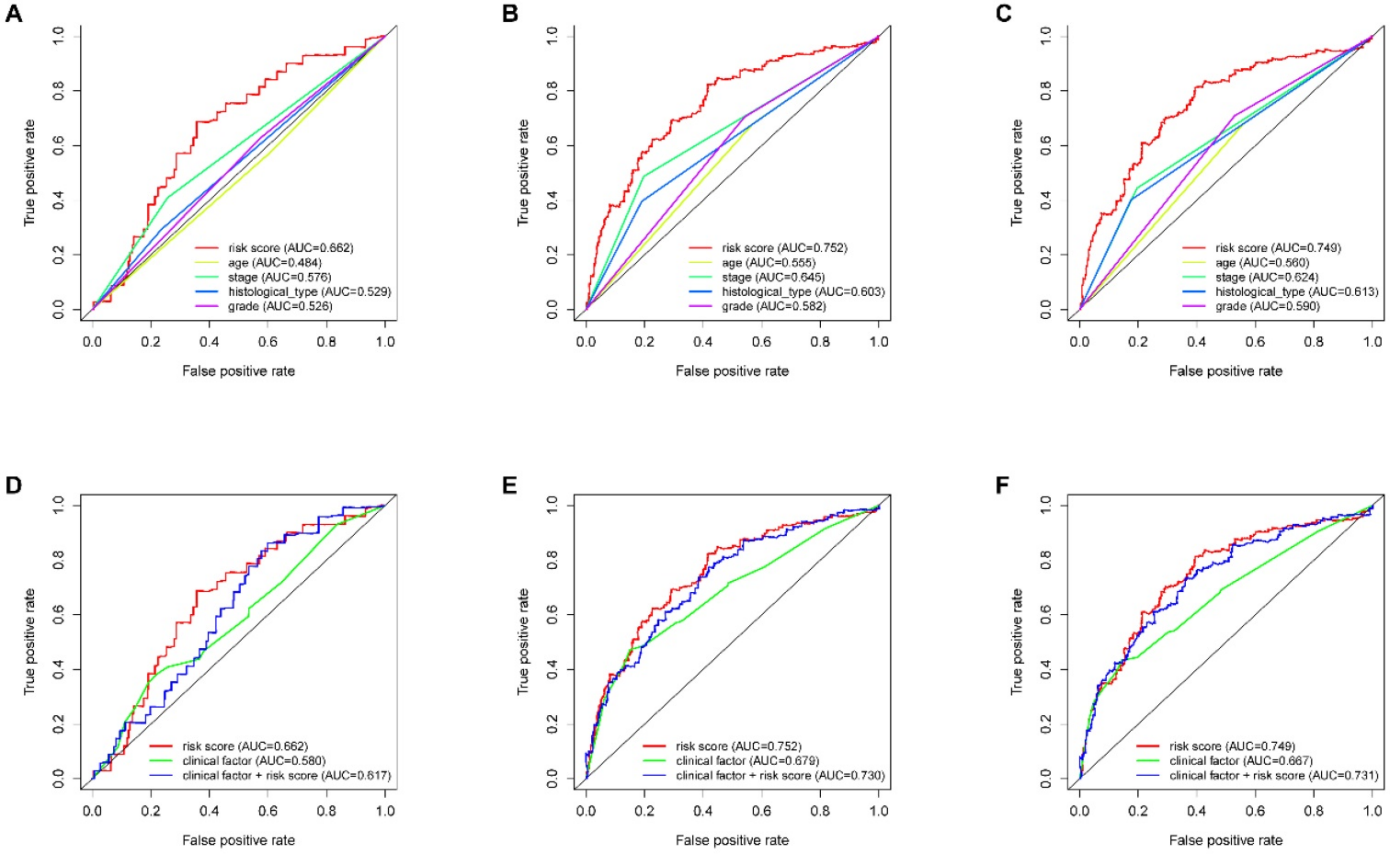
ROC analysis of clinical factors and the risk score. Calculated AUCs for risk score, age, stage, histological type, and grade of the total survival risk score according to the ROC curve at (A) one year, (B) three years, and (C) five years. Calculated AUCs for risk score and combined clinical factors of the total survival risk score according to the ROC curve at (D) one year, (E) three years, and (F) five years.

According to the median risk score, all EnCa samples were divided into high- and low-risk groups. The distribution of risk score, survival status, and expression of 13 hub lncRNAs in the training set are shown in Fig. 1A-B. Kaplan-Meier analysis suggested a remarkable difference between the two groups (*P*<0.001, Fig. 1C). In the training set, the 1-, 2-, 3-, and 5-year AUCs were 0.668, 0.878, and 0.884, respectively (Fig. 1D). To validate the prognostic value of this risk signature, the testing and entire cohort were used to further test the prognostic impact of the risk signature. The distributions of risk score, survival status, and expression of 13 hub lncRNAs in the testing and entire sets are shown in Fig. 2A-B and Fig. 3A-B. Similar to the training set, the clinical outcome of patients with high risk was remarkably worse than that of patients with low risk in both the testing (*P*=0.005) and entire sets (*P*<0.001) (Figs. 2C, 3C). Additionally, time-dependent ROC analysis demonstrated the satisfactory prognostic accuracy of the established signature in the testing and entire sets (Figs. 2D, 3D). Furthermore, among multiple clinicopathological factors, the risk score showed the largest AUC for 1-year, 3-year, and 5-year DFS (Fig. 4A-F).

### Expression of 13 hub lncRNAs and their association with immune cell infiltration

A total of 13 hub lncRNAs were incorporated into the construction of the novel immune-related prognostic signature, so we next determined their expression and association with immune cell infiltration in EnCa. As shown in Fig. S2, four lncRNAs, namely, AL080317.2, ZNF433-AS1, SCARNA9, and AC073046.1, were significantly overexpressed in EnCa tissues compared with normal tissues (*P*<0.05, Fig. S2A-D). Three lncRNAs, namely, AC016877.3, AC120053.1, and AL157932.1, were downregulated in EnCa tissues (*P*<0.05, Fig. S2E-G). However, the other six lncRNAs showed no remarkable dysregulation in tumor tissues (*P*>0.05, Fig. S3A-F). We further assessed the association between the expression of 13 hub lncRNAs and immune cell infiltration. Most lncRNAs were correlated with specific immune cell infiltration; among them, ERICH6-AS1 had the strongest correlation with immune cell infiltration (Fig. S4).

Immune-related mRNAs showing a Pearson correlation of > 0.5 with a 13-lncRNA signature were selected according to their association with lncRNAs, and altogether, 21 immune-related mRNAs were plotted into the network (Fig. S5A-B).

### Identification of risk score-associated biological pathways

To define the potential biological processes associated with risk score in EnCa, GSEA was conducted to identify the enriched KEGG pathways. The results showed that “cell cycle”, “ECM receptor interaction”, “ERBB signaling pathway”, “TGF beta signaling pathway”, and “Wnt signaling pathway” were enriched in EnCa samples with high-risk scores (Fig. S6A). Since these lncRNAs were immune-related genes, the low-risk group was enriched in several immune-related pathways, including “cytokine-cytokine receptor interaction” and “intestinal immune network for IgA production”, which were related to immunity (Fig. S6B). In addition, “Allograft rejection”, “Asthma”, and “Graft versus host disease” were also enriched in EnCa samples with low-risk scores (Fig. S6B).

### Survival analysis of the signature in the training, testing and entire sets

Based on the newly developed signature, we next assessed the prognostic impact of the risk signature in EnCa patients with different clinicopathological characteristics in the entire cohort. First, patients who were older (aged > 60 years), had poor differentiation (G3&G4), and had the mixed and serous subtype tended to have higher risk scores (*P*<0.05, Fig. S7A-C). Moreover, as shown in Fig. S8, the risk score reached satisfactory prognostic discrimination in all patients with different characteristics (*P*<0.05, Fig. S8A-D).

**Figure 5:**
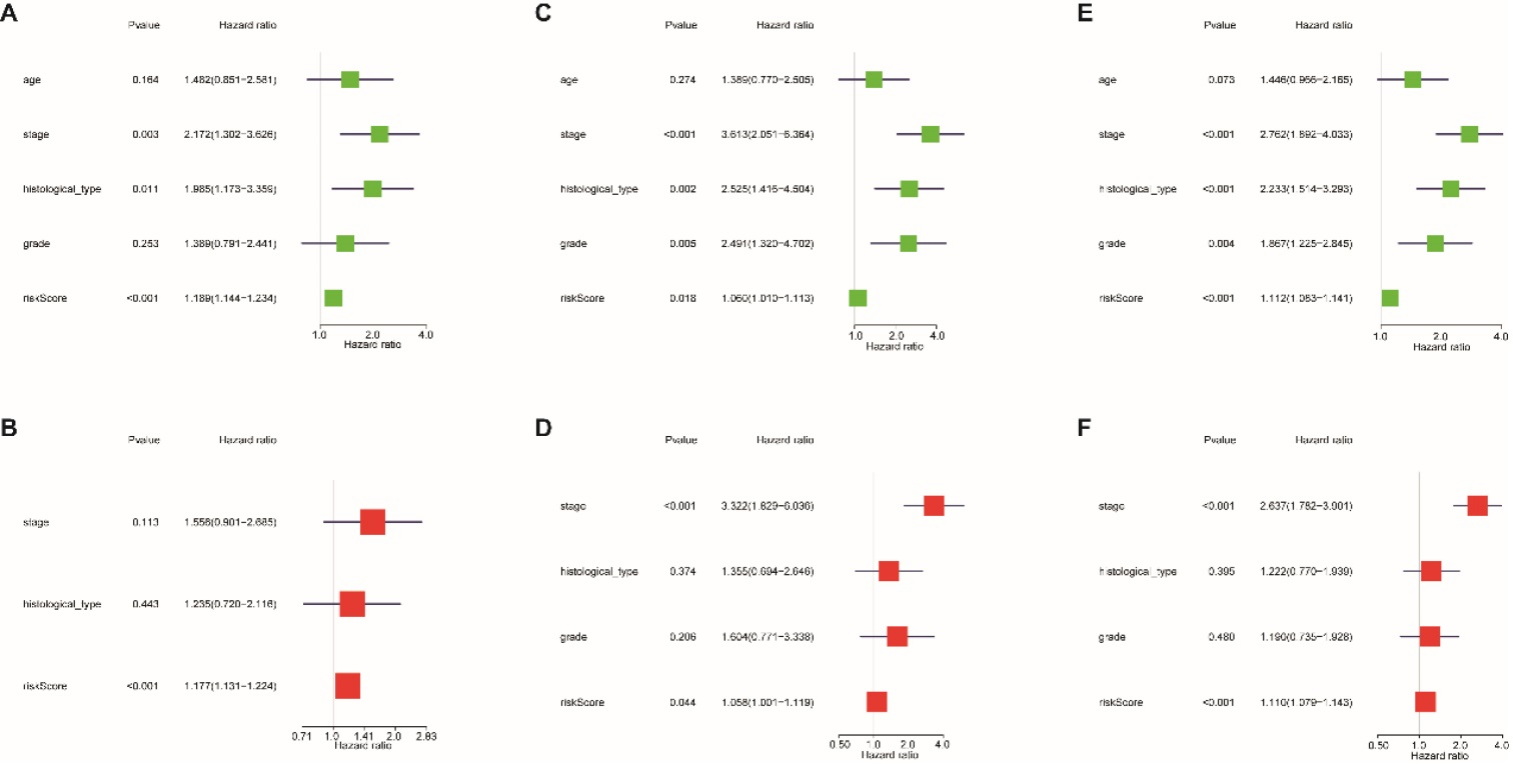
Cox regression analysis was used to assess the independent prognostic value of the risk score. Univariate Cox regression analysis of age, stage, histological type, grade, and risk score in the (A) training cohort, (B) testing cohort, and (C) entire cohort. The multivariate Cox regression analysis of age, stage, histological type, grade, and risk score in the (D) training cohort, (E) testing cohort, and (F) entire cohort.

Univariate and multivariate Cox regression analyses were used to evaluate whether the 13-lncRNA signature was an independent prognostic indicator for EnCa patients. In the univariate Cox regression analysis, the hazard ratios (HRs) of the risk score and 95% CIs were 1.189 (1.144-1.234), 1.060 (1.010-1.113), and 1.112 (1.083-1.141) in the training, testing, and entire sets, respectively (Fig. 5A-C). In the multivariate Cox regression analysis, the HRs of the risk score and 95% CIs were 1.177 (1.131-1.224), 1.058 (1.001-1.119), and 1.110 (1.079-1.143) in the training, testing, and entire sets, respectively (Fig. 5D-F).

**Figure 6:**
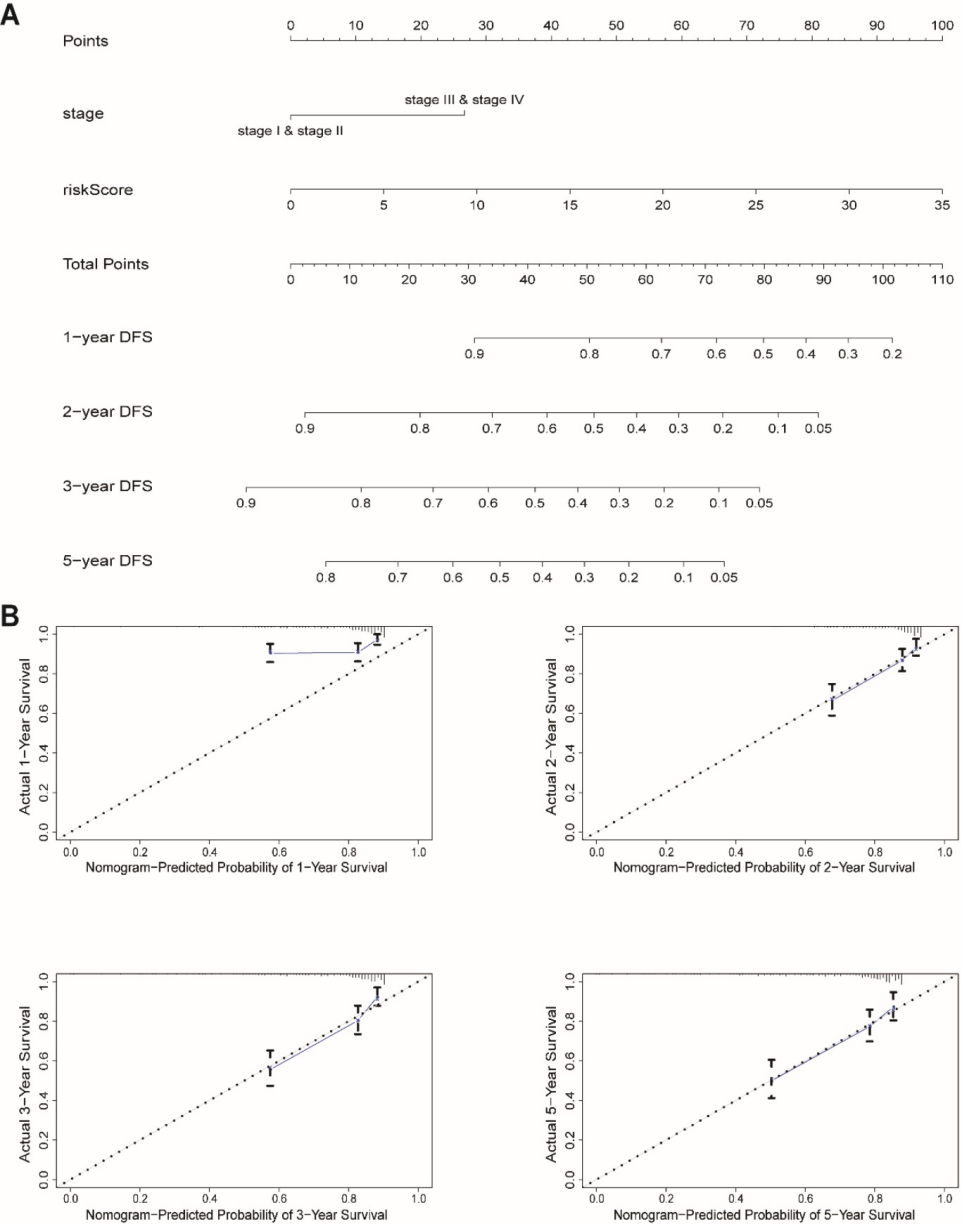
Construction of a nomogram for survival prediction. (A) The nomogram combining the signature with clinicopathological features. (B) Calibration plot showing that nomogram-predicted survival probabilities corresponded closely to the actual observed proportions.

We next built a nomogram to indicate 1-, 2-, 3-, and 5-year DFS among EnCa patients using stage and risk score, which was identified as an independent prognostic factor for EnCa patients (Fig. 6A). Calibration plots showed that the mortality estimated by the nomogram was close to the actual mortality (Fig. 6B). All findings suggested that the 13-lncRNA prognostic signature for EnCa was highly reliable.

### Different immune status between high‑ and low‑risk groups

Given that the 13-lncRNA signature was developed using immune-related lncRNAs, we further evaluated whether the novel signature was correlated with the expression of immune checkpoints. The results showed that patients with low risk tended to express higher CTLA-4 and PD-1 levels (*P*<0.05, Fig. S9A). In addition, the risk score was negatively correlated with the expression of CTLA-4 and PD-1 (*P*<0.05, Fig. S9B-D).

**Figure 7:**
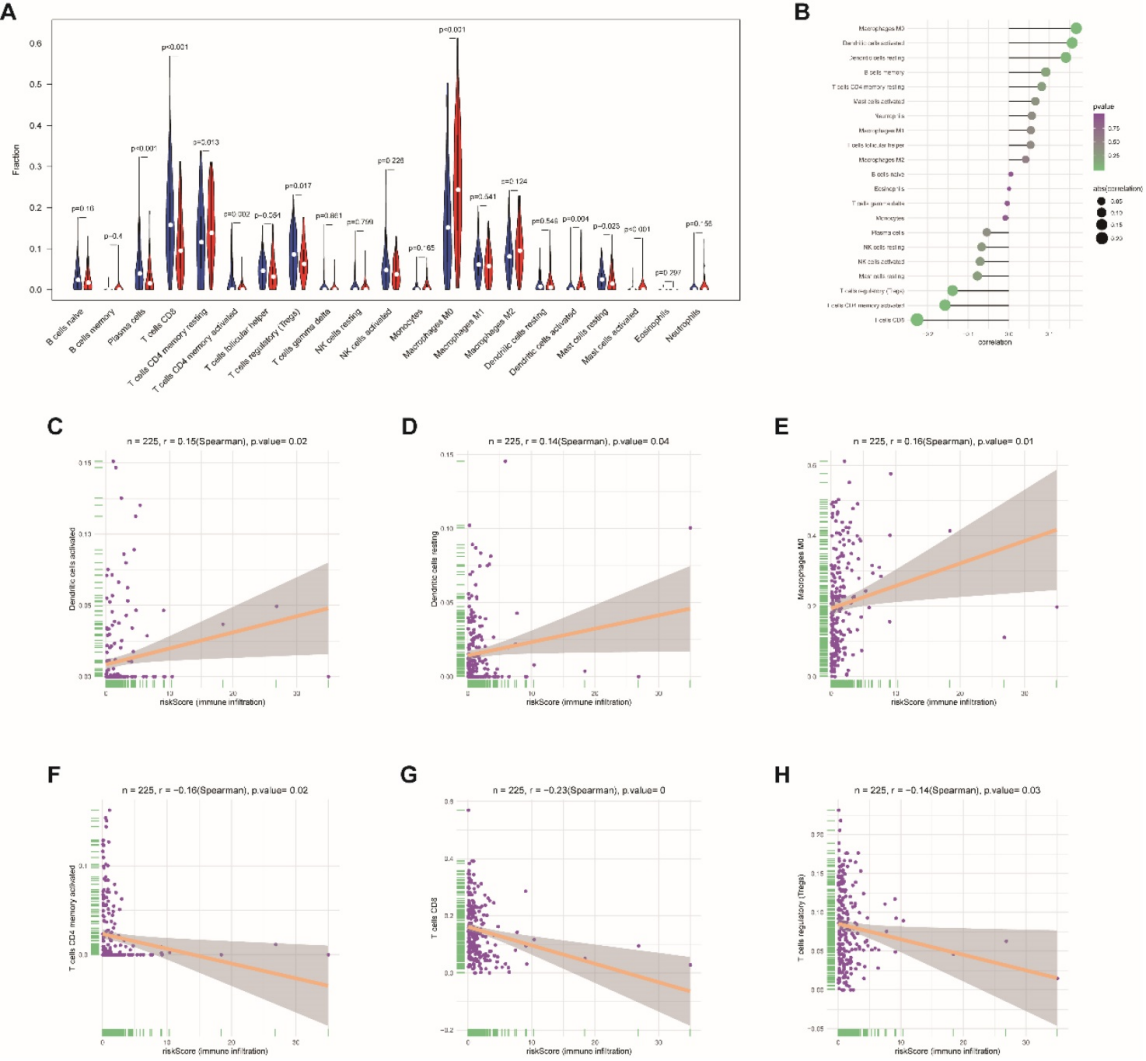
Association between immune cell infiltration and the immune-related risk signature. (A) The violin plot represents immune cell infiltration in the high- and low-risk groups. (B) The Bubble plot represents the correlation between immune cell infiltration and the immune-related risk score. Positive correlation between (C) activated dendritic cells, (D) resting dendritic cells, or (E) M0 macrophage infiltration and the immune-related risk score. Negative correlation between (F) activated CD4+ memory T cells, (G) CD8+ T cells, or (H) regulatory T cell infiltration and the immune-related risk score.

Moreover, to assess the correlations between risk score and tumor immune cell infiltration, CIBERSORT was applied to estimate the proportion of the 22 immune cells by using transcriptome data in each EnCa sample. We appraised that resting CD4+ memory T cells, M0 macrophages, and activated master cells were positively correlated with the 13-lncRNA risk score, while plasma cells, CD8+ T-cells, activated CD4+ memory T cells, regulatory T cells, activated dendritic cells, and resting master cells were negatively correlated with the 13-lncRNA risk score (*P*<0.05, Fig. 7A-B). In addition, the top 3 immune cells that were most positively and negatively associated with the risk score (*P*<0.05) are shown in Fig. 7C-H.

### Mutational loading in high- and low‑risk groups

**Figure 8:**
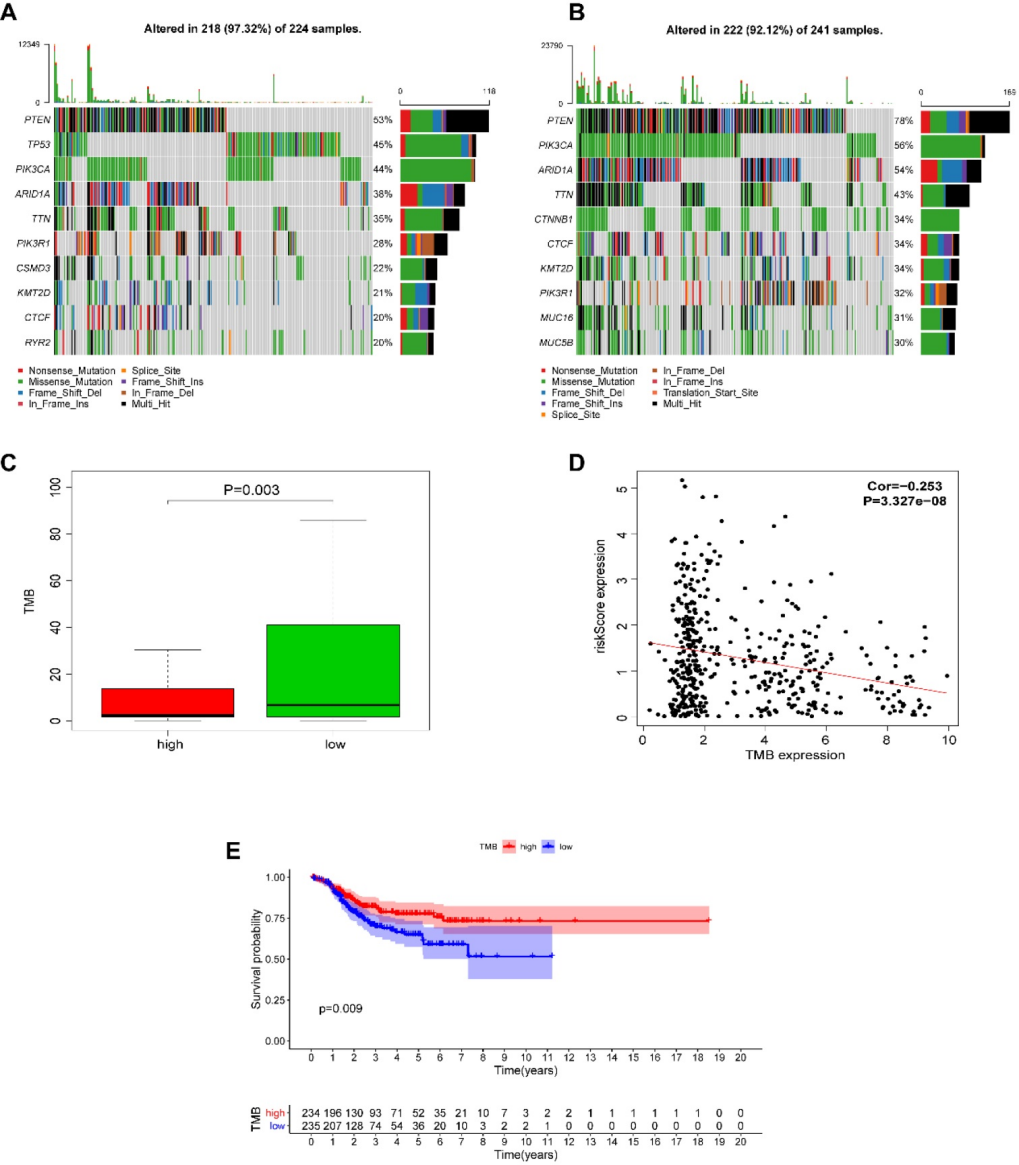
Alteration landscape for high- and low-risk EnCa samples in the TCGA cohort. (A, B) The rates of PTEN mutation, PIK3CA mutation, ARID1A mutation, TTN mutation, and PIK3R1 mutation in EnCa with a high-risk score were lower than those with EnCa with a low-risk score. (C) EnCa patients with high-risk scores had a heavier tumor mutation burden than with those with low-risk scores. (D) Negative correlation between tumor mutation burden and the immune-related risk score. (E) Kaplan-Meier curve analysis of DFS of EnCa patients with different tumor mutation burdens.

We next investigated whether EnCa with a high-risk score was associated with tumor mutation burden (TMB). The altered landscapes in EnCa with high- or low-risk scores are shown in Fig. 8A-B. Ten genes were mutated in >20% of samples with high-risk scores: PTEN (53%), TP53 (45%), PIK3CA (44%), ARID1A (38%), TTN (35%), PIK3R1 (28%), CSMD3 (22%), KMT2D (21%), CTCF (20%), and RYR2 (20%). Ten genes were mutated in >30% of samples with low-risk scores: PTEN (78%), PIK3CA (56%), ARID1A (54%), TTN (43%), CTNNB1 (34%), CTCF (34%), KMT2D (34%), PIK3R1 (32%), MUC16 (31%), and MUC5B (30%). Specifically, the rates of PTEN mutation, PIK3CA mutation, ARID1A mutation, TTN mutation, PIK3R1 mutation, KMY2D mutation, and CTCF mutation were lower in EnCa with high-risk scores than in EnCa with low-risk scores (Fig. 8A-B). In addition, patients with low-risk scores tended to have a higher TMB than those with high-risk scores (*P*=0.003, Fig. 8C). Moreover, the risk score was negatively correlated with TMB (*P*<0.001, Fig. 8D). Furthermore, we found that high TMB was associated with poorer DFS (*P*=0.009, Fig. 8E).

### Response to immunotherapy and chemotherapy in high‑ and low‑risk groups

We also assessed the association between IPS and our immune signature score. The IPS, PD1-PD-L1-PD-L2-IPS, CTLA4-IPS, and PD1-PD-L1-PD-L2-CTLA4-IPS scores were calculated to evaluate the potential for patients to be placed on immunotherapy. The results showed that the PD1-PD-L1-PD-L2-CTLA4-IPS score was notably higher in the low-risk group (*P*<0.05), while the other three scores had no significant association with the risk score (Fig. S10).

Because chemotherapy is the standard therapeutic strategy for EnCa patients, we assessed the response of patients in these two groups to several common chemo drugs. We estimated the IC50 for each sample in the TCGA dataset based on the predictive model of these chemo drugs. We observed a significant difference in the estimated IC50 between the low- and high-risk groups for gemcitabine, vinblastine, and vorinostat (*P*<0.05), indicating that patients with high risk could be more resistant to these chemotherapies, which explains the poor prognosis of patients in the high-risk group (Fig. S11).

## Discussion

EnCa is a common gynecological malignancy that poses a great threat to women's lives. Although more than 70% of cases can be diagnosed at the early stage, approximately 30% of patients have regional and/or distant metastasis when first seen in the clinic 20, 21. Unfortunately, the prognosis of EnCa patients with advanced-stage disease is often unsatisfactory. The prognostic evaluation of EnCa has always been a hot topic for concerned scholars. Construction of scoring models will contribute to quantification of the prognostic evaluation criteria, and an increasing number of studies are successfully establishing precedents in this regard 22-24.

In the current research, a series of systematic bioinformatics analyses were conducted, ultimately leading to the development of a novel 13-lncRNA signature associated with the clinical outcome of EnCa patients; this signature included AL080317.2, ERICH6-AS1, AC016877.3, MCCC1-AS1, AC120053.1, AC138932.5, ZNF433-AS1, SCARNA9, DBH-AS1, AL157932.1, AC073046.1, POC1B-AS1, and AP003419.3. The novel signature constructed based on the expression of these 13 immune-related lncRNAs allowed us to distinguish samples with different risks with relatively high accuracy. In the training set, EnCa patients in the high-risk group had a shorter DFS than those in the low-risk group, and consistent findings were obtained in the testing and entire sets. Moreover, univariate and multivariate Cox regression analyses confirmed that the signature was an independent factor for predicting DFS in EnCa. All findings confirmed that the 13-lncRNA prognostic model could accurately predict the DFS of patients with EnCa.

Among the 13 immune-related lncRNAs, ERICH6-AS1, MCCC1-AS1, AC120053.1, AC138932.5, ZNF433-AS1, DBH-AS1, AL157932.1, AC073046.1, POC1B-AS1, and AP003419.3 were risk-associated lncRNAs, while AC016877.3, AL080317.2, and SCARNA9 were protective lncRNAs. LncRNA-mRNA coexpression analysis was conducted to evaluate the functions of related lncRNAs. Several lncRNAs have been identified as functional genes in cancers. For example, DBH-AS1 may act as an oncogene, and downregulation of DBH-AS1 predicts better prognosis and suppresses osteosarcoma progression by inhibiting the PI3K/AKT pathway 25. In addition, DBH-AS1 can accelerate the tumorigenesis and development of hepatocellular carcinoma by sponging miR-138 and regulating the FAK/Src/ERK pathway 26. Wang *et al.* developed of a multi-RNA-type-based model for recurrence-free survival (RFS) evaluations of patients with EnCa 27. In addition, we also determined the expression of lncRNAs in normal and tumor tissues. Four lncRNAs were upregulated, while three lncRNAs were downregulated. Therefore, the 13 immune-related lncRNAs might be potential therapeutic targets because of the dysregulation of lncRNAs.

In recent years, immunotherapy has been considered a novel therapeutic strategy for EnCa 4, 5, 28. The tumor microenvironment (TME), which contains extracellular matrix, fibroblasts, endothelial cells, and multiple immune cells, plays a critical role in tumor progression, immune escape and responses to therapies, especially immunotherapies 29. Thus, identification of a single gene and/or gene signature that correlates with immune cell infiltration is essential for the assessment of responses to immunotherapies.

As an important part of the current research, we evaluated the association between the expression of 13 hub lncRNAs and immune cell infiltration. Most lncRNAs were correlated with specific immune cell infiltration; among them, ERICH6-AS1 had the strongest correlation with immune cell infiltration. In addition, the risk model based on the expression of 13 immune-related lncRNAs was also significantly associated with different immune statuses in EnCa. TMB is an emerging biomarker for assessing the response to PD-1/PD-L1 inhibitors 30, 31. We also evaluated the correlation between the risk score of the prognosis signature and TMB. Patients with low risk tended to have a higher TMB than those with high risk, and the risk score was negatively correlated with TMB. Overall, the 13-lncRNA signature might serve as a biomarker to assess the feasibility of immunotherapies.

Although immunotherapy could be a novel therapeutic strategy, EnCa is usually treated with a combined regimen of surgery and chemotherapy 32, 33. Using the GDSC database, we found that EnCa patients with low risk could be more sensitive to commonly used chemotherapies, including gemcitabine, vinblastine, and vorinostat, than those with high risk, which demonstrated that low-risk patients may benefit from this combination of chemotherapy.

However, the current research has several unavoidable limitations. Most important of all, the research is based on bioinformatics analysis, and there is no external EnCa data as a validation set; secondly, there were no recruited cohorts for verification of prognostic value of the signature in EnCa and response to immunotherapy and chemotherapy in high‑ and low‑risk groups. Whatever, we will pay more attention to emerging external data for further verifying our model in the future.

## Conclusions

In conclusion, we identified immune-related lncRNAs in EnCa and developed a 13-lncRNA signature that has significant prognostic value for patients with EnCa. Furthermore, high- and low-risk groups, which were divided based on the median risk score, displayed different immune statuses and responses to immunotherapy and chemotherapy.

## Supplementary Material

Supplementary figures and tables.

## Abbreviations

EnCa: endometrial carcinoma
FIGO: International Federation of Gynecology and Obstetrics
LncRNAs: long noncoding RNAs
PD-L1: programmed cell death 1 ligand 1
DFS: disease-free survival
TMB: tumor mutation burden
GSEA: gene set enrichment analysis
MAF: mutation annotation format
IPS: immunophenoscore
TCIA: Cancer Immunome Atlas
GDSC: Genomics of Drug Sensitivity in Cancer
IC50: half-maximal inhibitory concentration
RFS: recurrence-free survival
TME: tumor microenvironment
