# Supplementary material for: Development of a novel immune-related lncRNA signature as a prognostic classifier for endometrial carcinoma
Source: Int J Biol Sci. 2021 Jan 1;17(2):448–59. doi: 10.7150/ijbs.51207 (PMC7893582; doi:10.7150/ijbs.51207)
Supplement: Supplementary file 1 — Supplementary figures and tables. [file ijbsv17p0448s1.pdf]

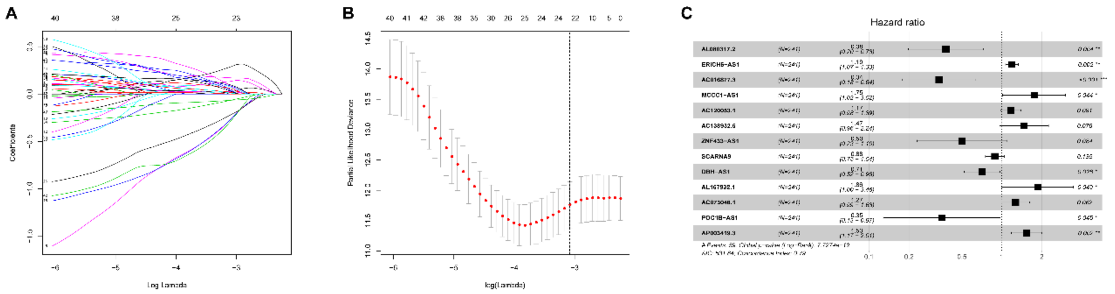

Figure S1. LASSO Cox regression analysis of immune-related lncRNAs. (A) LASSO coefficient profiles of immune-related lncRNAs. (B) 10-fold cross-validation results that identified optimal values of the penalty parameter λ. (C) The HR and P value from the univariable Cox HR regression of 13 lncRNAs.

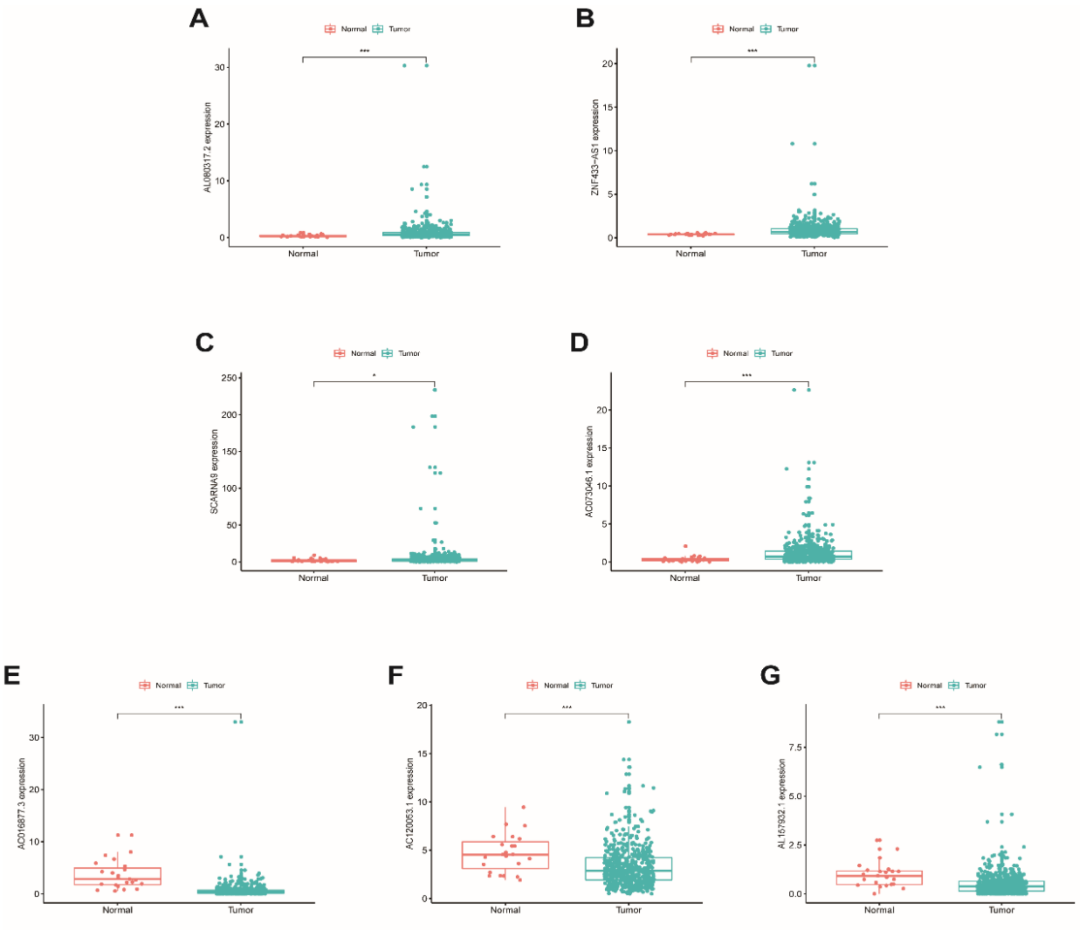

Figure S2. Expression of immune-related lncRNAs in EnCa and normal tissues (lncRNAs with differential expression). (A) AL080317.2. (B) ZNF433-AS1. (C) SCARNA9. (D) AC073046.1. (E) AC016877.3. (F) AC120053.1. (G) AL157932.1.

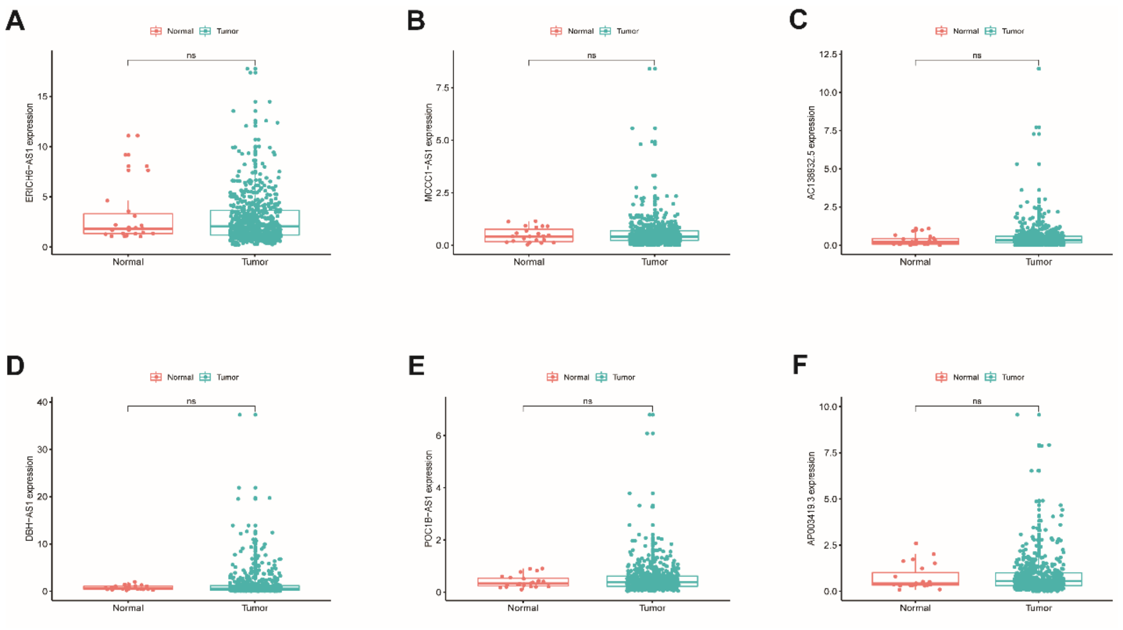

Figure S3. Expression of immune-related lncRNAs in EnCa and normal tissues (lncRNAs without differential expression). (A) ERICH6-AS1, (B) MCCC1-AS1, (C) AC138932.5, (D) DBH-AS1, (E) POC1B-AS1, (F) AP003419.3.

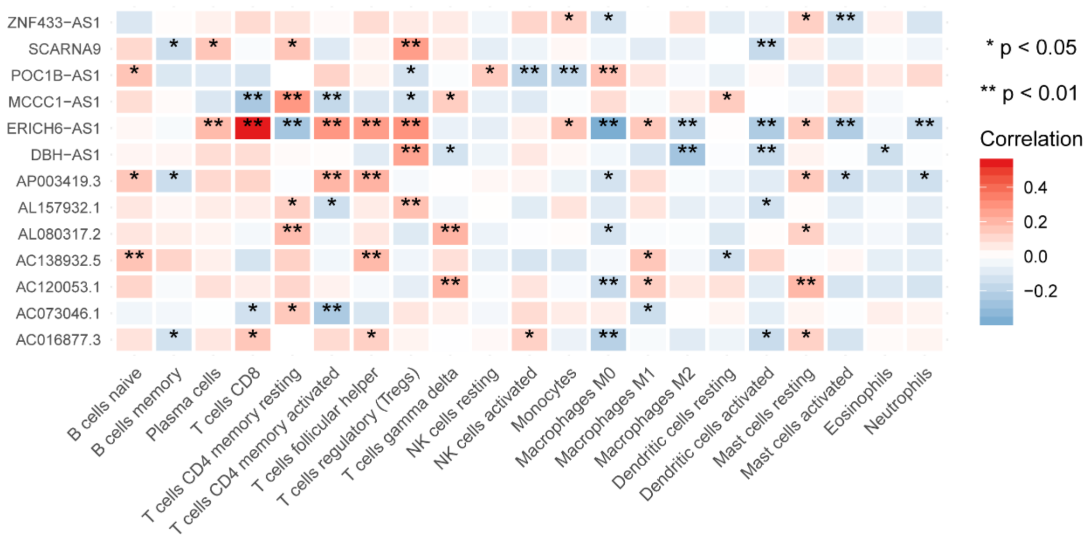

Figure S4. Correlation of expression of 13 immune-related lncRNAs and infiltration levels of immune cells.

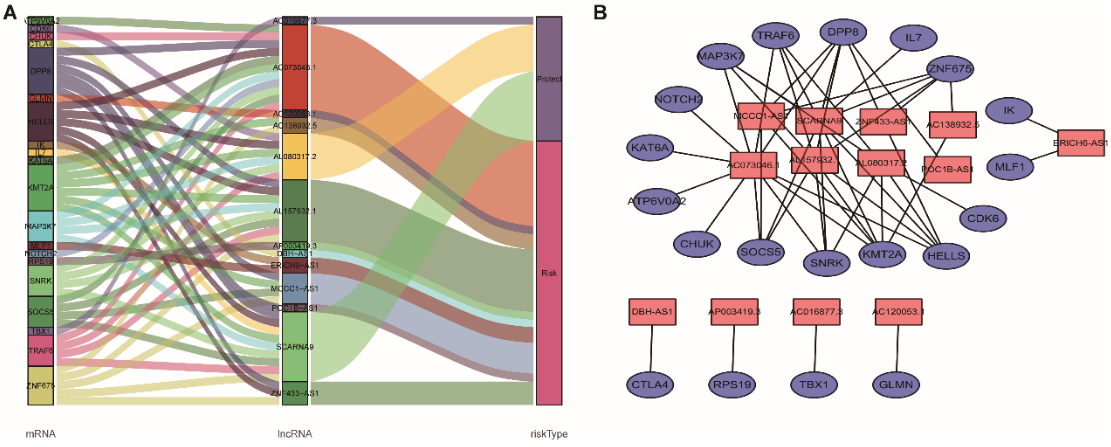

Figure S5. Network between 13 immune-related lncRNAs and related functional genes. (A) Sankey diagram representing associations between functional genes, immune-related lncRNAs and risk types. (B) LncRNA-mRNA network of 13 immune-related lncRNAs and related functional genes.

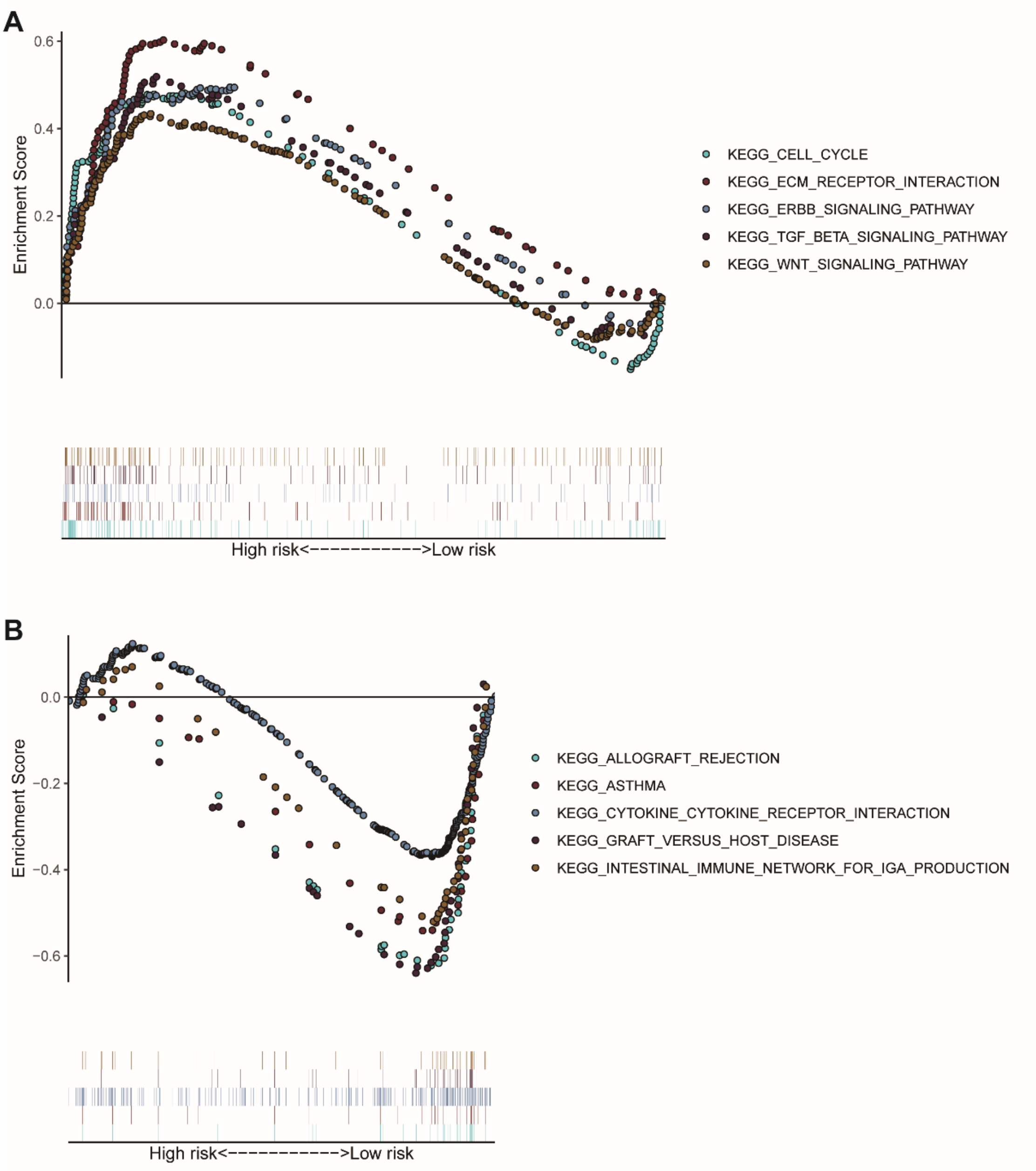

Figure S6. GSEA using the TCGA dataset. (A) The five most functional gene sets enriched in EnCa samples with high risk scores. (B) The five most functional gene sets enriched in EnCa samples with low risk scores.

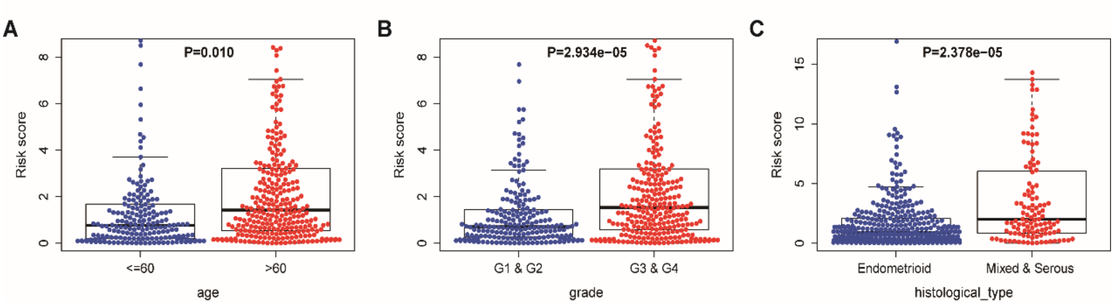

Figure S7. Risk score distribution in EnCa patients with different clinical features. (A) EnCa patients of different ages. (B) EnCa patients with different grades. (C) EnCa patients with different histological types.

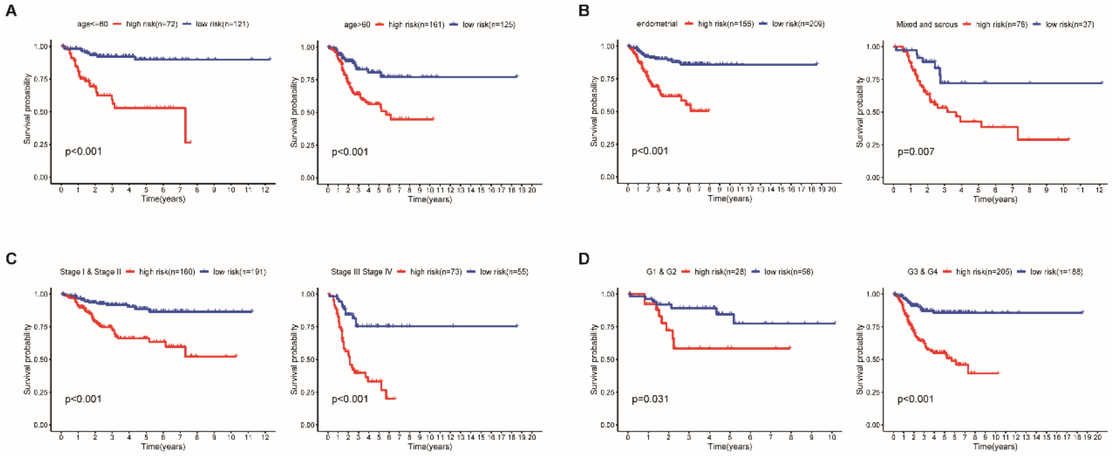

Figure S8. Subgroup analysis of prognostic value of risk score in EnCa. (A) Prognostic value of risk score in patients of different ages. (B) Prognostic value of risk score in patients with different histological types. (C) Prognostic value of risk score in patients with different stages. (D) Prognostic value of risk score in patients with different grades.

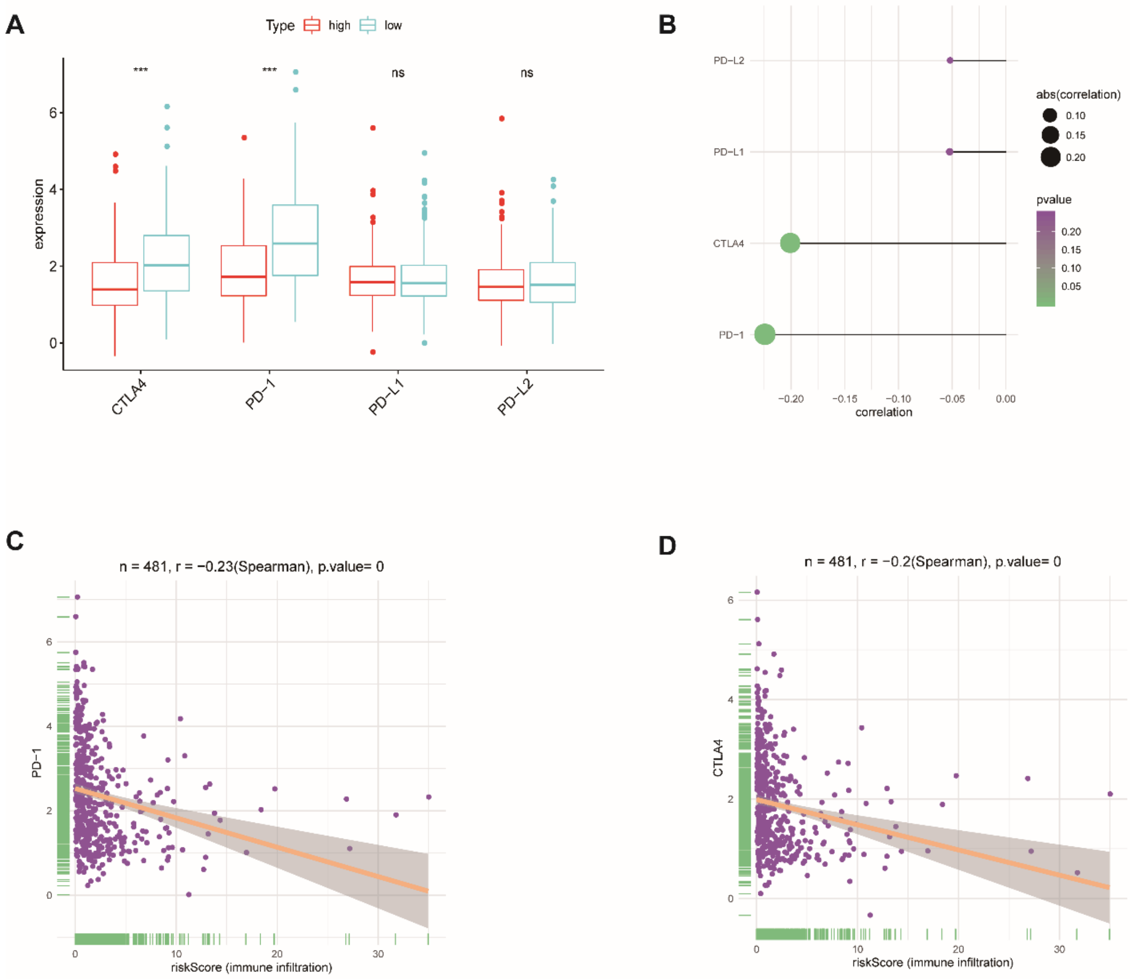

Figure S9. Association between immune checkpoint expression and the immune-related risk signature. (A) The box plot represents immune checkpoint expression in the high- and low-risk groups. (B) The bubble plot represents the correlation between immune checkpoint expression and the immune-related risk score. Negative correlation between (C) PD-1 or (D) CTLA-4 expression and the immune-related risk score.

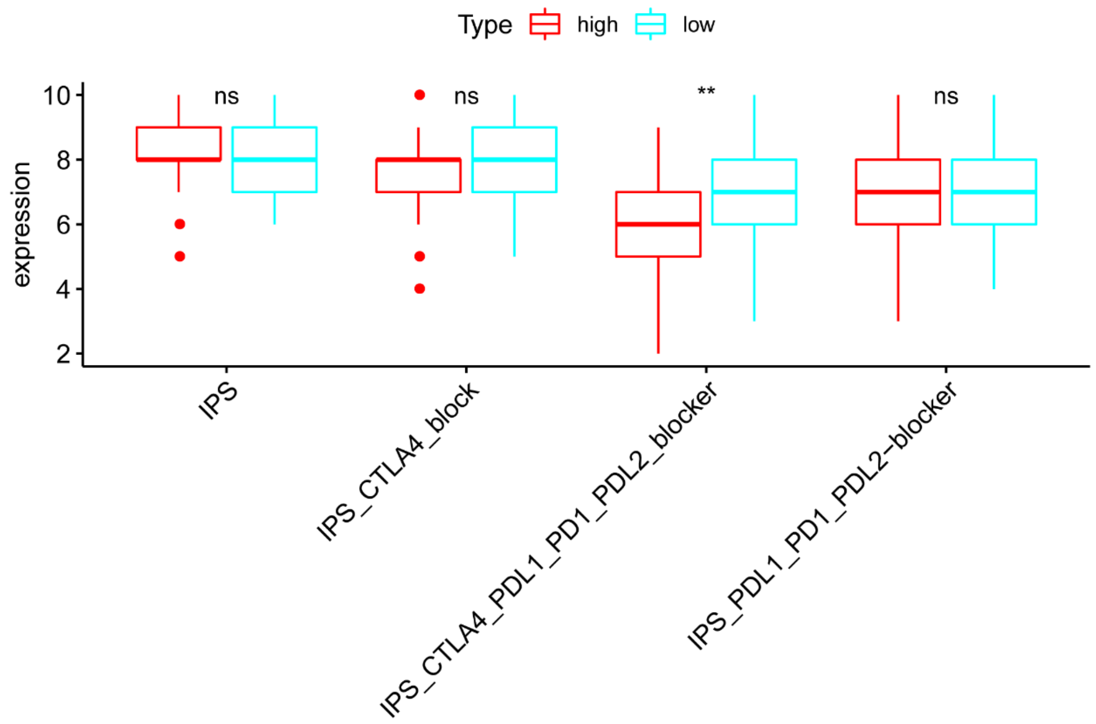

Figure S10. Association between IPS and the immune-related risk signature.

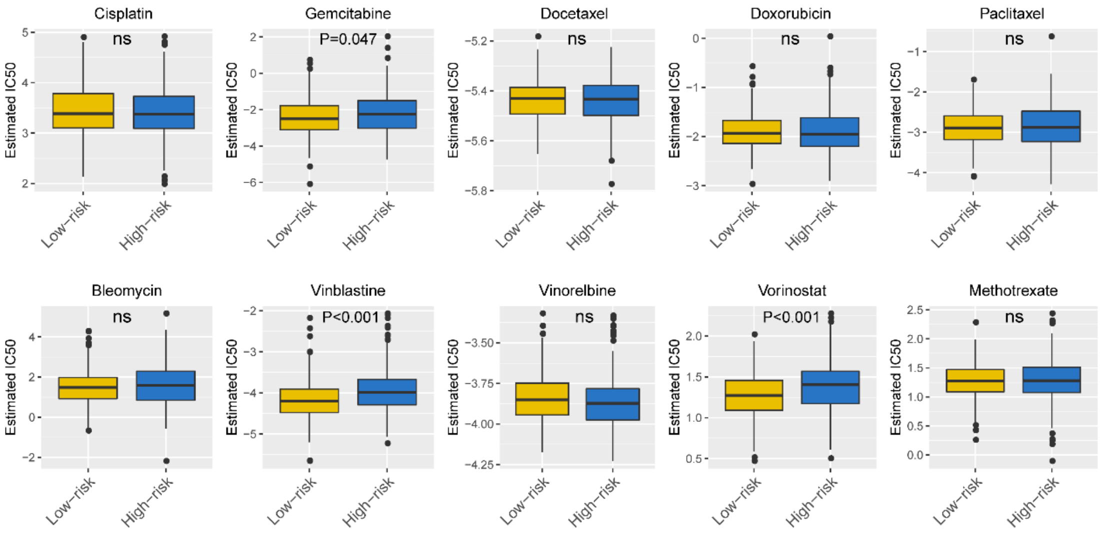

Figure S11. Association between risk score and chemosensitivity in EnCa.
